# A Case Report of the Synchronous Occurrence of Ovarian Granulosa Cell Tumour and Malignant Endometrial Polyp with Immunohistochemical Expression of Hormone Receptors and Biomarkers p-53 and Ki-67

**DOI:** 10.3390/reports7040103

**Published:** 2024-11-20

**Authors:** Krum Vladov, Ekaterina Uchikova, Maria Koleva-Ivanova, Kamen Yamakov, Veselin Belovezhdov, Gita Yamakova-Vladova, Eleonora Hristova-Atanasova

**Affiliations:** 1 Clinic of Obstetrics and Gynecology, University Hospital “St. George”, 4000 Plovdiv, Bulgaria; ekaterina.uchikova@mu-plovdiv.bg (E.U.); kamen.yamakov@mu-plovdiv.bg (K.Y.); gita.vladova@mu-plovdiv.bg (G.Y.-V.); 2 Department of Obstetrics and Gynecology, Faculty of Medicine, Medical University of Plovdiv, 4000 Plovdiv, Bulgaria; 3 Department of General and Clinical Pathology, Faculty of Medicine, Medical University of Plovdiv, 4000 Plovdiv, Bulgaria; mariya.koleva@mu-plovdiv.bg (M.K.-I.); veselin.belovezhdov@mu-plovdiv.bg (V.B.); 4 Department of Social Medicine and Public Health, Faculty of Public Health, Medical University of Plovdiv, 4000 Plovdiv, Bulgaria

**Keywords:** abnormal uterine bleeding, endometrial polyps, ovarian tumours, oestrogen receptor, progesterone receptor

## Abstract

**Background and Clinical Significance:** Abnormal uterine bleeding during the postmenopausal years is a pathological sign that may be due to simultaneous intrauterine and ovarian pathology. Granulosa cell tumours of the ovary are malignant neoplasms producing oestradiol, which leads to the abnormal proliferation of the endometrium, precancerous lesions, and endometrial carcinoma type I. **Case Presentation:** The authors present a clinical case of a 67-year-old woman with postmenopausal bleeding who underwent a total abdominal hysterectomy with bilateral adnexectomy, pelvic lymphadenectomy, and partial omentectomy. The histopathological examination showed a granulosa cell adult-type ovarian tumour and a malignant endometrial polyp with atypical hyperplasia of the endometrium. **Conclusions:** The immunohistochemical analysis of the malignant endometrial polyp confirmed the expression of oestrogen, progesterone receptors, and the biomarker Ki-67.

## 1. Introduction

Abnormal uterine bleeding is more often associated with intrauterine lesions or hormonal imbalances. From a pathogenetic point of view, hyperestrogenemia is the leading factor [1]. Endometrial polyps (EPs) are benign neoplasms of the endometrium that are diagnosed at any age and in 20% of postmenopausal women, which can cause bleeding. In a small number of cases, polyps can transform into endometrioid adenocarcinoma, which is categorised as type I endometrial carcinoma. This type arises from precursor lesions and under the influence of endogenous or exogenous oestrogens as a multistage progression from endometrial hyperplasia without atypia to the appearance of atypia (AEH). Type II non-endometrioid carcinoma arises de novo without a precursor lesion and is oestrogen-independent [2,3]. EP undergo malignant transformation in a wide range of up to 12.9% under the influence of risk factors such as advanced age, hyperestrogenemia, postmenopausal bleeding, obesity, and the use of tamoxifen [4,5]. The purpose of this case report is to assess the impact of a hormone-producing tumour on an endometrial polyp. Granulosa cell tumours lead to hyperestrogenemia. The simultaneous presence of a hormone-producing ovarian tumour is also possible: a granulosa cell ovarian tumour leads to hyperestrogenemia with a proliferative effect on the endometrium with subsequent precancerous and malignant transformation. The production of oestradiol by granulosa cell tumours is the leading cause of the development of endometrial hyperplasia in 25% to 50% of the cases, of which 5% to 13% are associated with highly differentiated early-stage endometrioid adenocarcinoma [6]. The exact diagnosis is based on clinical imaging and histological diagnostic methods, and performing immunohistochemistry provides clarity on the etiopathogenesis of endometrial polyps. This clinical case concerns a 67-year-old postmenopausal woman experiencing abnormal uterine bleeding. Following surgical treatment, the diagnosis revealed the simultaneous presence of an adult granulosa cell ovarian tumour, a malignant endometrial polyp, and atypical hyperplasia of the endometrium.

## 2. Case Presentation

### 2.1. Patient Information

The patient was a 67-year-old woman, gravida 2, para 2. She was urgently hospitalised on 10 February 2023 at the Obstetrics and Gynaecology Clinic of St. George University Hospital in Plovdiv, Bulgaria. Her complaints were of abnormal uterine bleeding with varying intensity for the past 7 days. She had been in menopause for 30 years without taking hormone replacement therapy. She had the following comorbidities: essential arterial hypertension and obesity, without a history of exposure to harmful factors in a professional environment or in her personal lifestyle.

### 2.2. Clinical Examination and Diagnostic Assessment

From the general physical status, there was no evidence of anaemia. Abdominal inspection and palpation ruled out signs of an acute abdomen. The body mass index was 30.1 kg/m^2^. A gynaecological examination with a speculum revealed a vagina stained with dark blood with clots and a slight descent of the anterior vaginal wall, a cylindrical vaginal portion of the cervix with a closed cervical canal, and scanty uterine bleeding. From the vaginoabdominal bimanual palpation, the uterus was in anteversion flexion, generally enlarged for the third lunar month, with a smooth surface, preserved mobility, and painlessness. On the left side, the adnexa were not palpable, but on the right, palpation revealed the presence of a solid painless tumour formation with an approximate size of about 5 cm and uneven swelling; the Pouch of Douglas was painless. The PAP smear (Papanicolaou test) was group II, which is normal. The tumour marker CA 125 was within the normal range of 32 U/mL from the reference range below 35 U/mL.

The complete blood count, biochemical parameters, coagulation, and iron studies were without deviation. A transvaginal ultrasonography (TVUS) was performed, in which the uterus was visualised with an anterior–posterior diameter of 6.35 cm. In the uterine cavity, there was a hypoechogenic endometrium of 2.88 cm. with a hypoechogenic shadow and, on the right, an ovarian tumour formation with a diameter of 5.92 cm. There was no free fluid present in the CD.

### 2.3. Management

**Figure 1:**
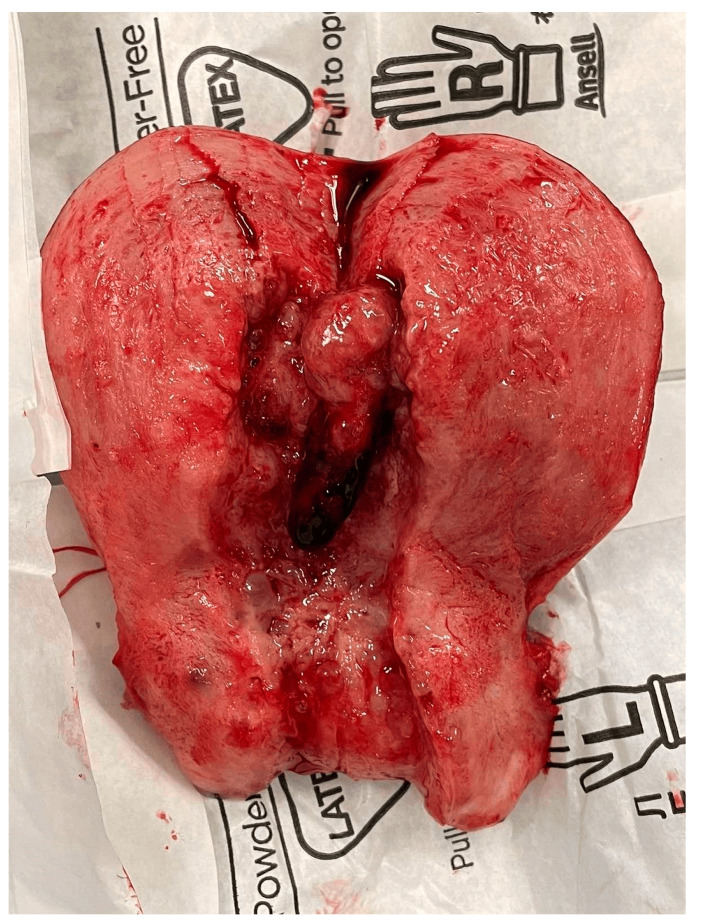
Sectional surface of the uterus with an endometrial polyp from the fundus of the uterus; histological result: malignant endometrial polyp.

After obtaining informed consent, surgical treatment was performed, consisting of a total abdominal hysterectomy with bilateral salpingo-oophorectomy, pelvic lymphadenectomy, and partial resection of the infracolic omentum. After performing a median laparotomy, a peritoneal lavage was taken for intraoperative cytological examination, which was positive for tumour cells. A tumour formation originating from the right ovary was found, located intraligamentary to the right broad ligament, with a solid cystic structure and a soft elastic consistency of the solid areas measuring 6 cm. The cut surface of the uterus revealed the presence of a polypoid formation in the uterine cavity originating from the fundus (Figure 1). An intraoperative frozen section confirmed a malignant tumour of the ovary. The total blood loss was 150 mL. After the result confirmed a granulosa cell tumour in the ovary, the oestradiol was examined in the serum, the value of which was in the reference interval at 19.8 pmol/L (18.4–505). The postoperative period of seven days was normal. The patient was discharged from the hospital with a strict recommendation to consult a psychologist, to have a CT scan performed, and to start chemotherapy according to the imaging data.

After the operative intervention in the Obstetrics and Gynaecology Clinic at St. George University Hospital, Plovdiv, Bulgaria, the patient was admitted to the Complex Oncology Centre for postoperative care. A CT was performed, and she underwent postoperative treatment with six courses of adjuvant chemotherapy with platinum-based agents. Until the end of 2023, she was followed up regularly every 3 months, and since the beginning of 2024 every 6 months. During this period, no recurrence of ovarian cancer has been detected. The patient adheres strictly to the dispensary regimen.

### 2.4. Histopathological Examination and Immunohistochemical Analysis of Malignant Endometrial Polyp

**Figure 2:**
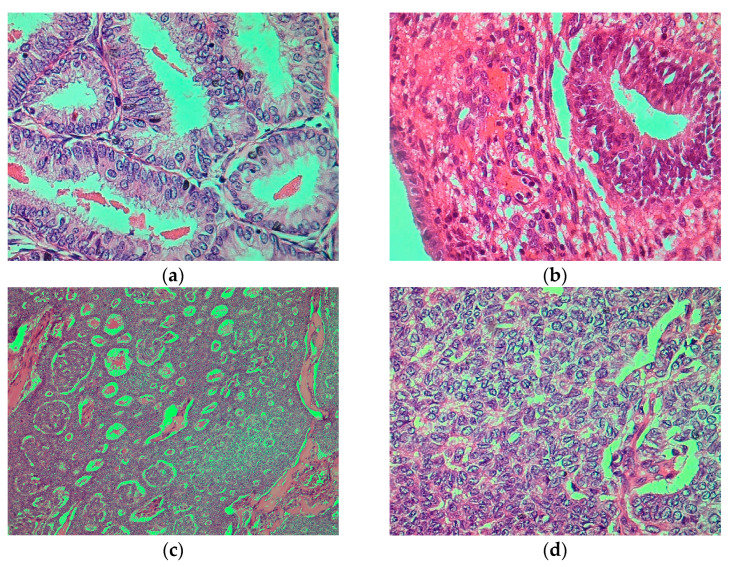
(**a**) Foci of endometrial atypical hyperplasia showing architectural features on low magnification: crowded aggregates with altered and branching glands. In these areas of AEH, the glands exceeded the stroma, haematoxylin–eosin, ×50. (**b**) Fragment of the endometrial polyp characterised by the presence of atypical hyperplastic features confined to the polyp, haematoxylin–eosin, ×50. (**c**) The tumour cells are arranged predominantly in a micropapillary pattern in which they surround small spaces containing eosinophilic material (Call–Exner bodies), haematoxylin–eosin, ×50. (**d**) The tumour cells have uniform pale round nuclei with nuclear grooves (coffee beans) and scant cytoplasm, haematoxylin–eosin, ×400.

Histopathological examination, conducted by staining the paraffin preparations with haematoxylin–eosin, confirmed atypical hyperplasia of the surrounding endometrium and a malignant endometrial polyp. The results are shown in Figure 2a–d.

**Figure 3:**
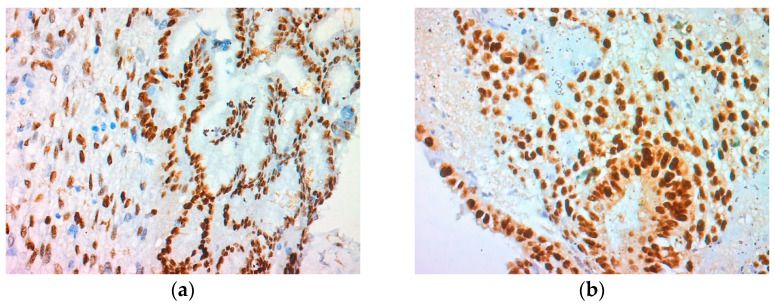

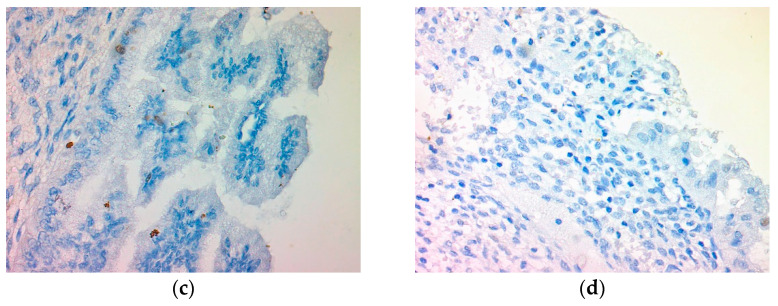
An additional immunohistochemical examination showed that the atypical hyperplastic glands confined to the polyp showed a strong expression for (**a**) ER, anti-ER ×100; (**b**) PR, anti-PR ×100; (**c**) the immunohistochemical examination presented a lack of expression of Ki-67, anti Ki-67 ×100; (**d**) the immunohistochemical examination presented a lack of expression of p-53, anti-p53 ×100.

The malignant endometrial polyp underwent the immunohistochemical analysis of the biomarkers Ki-67 and p-53, as well as the immunoexpression of the oestrogen and progesterone receptors (Figure 3). The epithelial and stromal cells had a moderate to strong intensity of the expression of oestrogen and progesterone receptors. The biomarker p-53 had a lack of expression, and Ki-67 had positive expression only in solitary epithelial cells.

## 3. Discussion

According to the WHO classification of ovarian tumours, adult and juvenile granulosa cell tumours of the ovary originate from the sexcord stromal cells. Their prevalence is about 2% of all ovarian tumours, with a peak age of incidence of 50–55 [7]. Granulosa cell tumours are characterised by the continuous and unimpeded secretion of oestrogen from the ovary, which has a proliferative effect on the endometrium [8].

Due to oestrogen secretion, abnormal uterine bleeding in the postmenopausal years is the most common manifestation of an adult-type tumour. Most of these are confined to the ovary without haematogenous and lymphogenic spread, which determines the 10-year survival rate of over 90% [9]. Hyperestrogenemia, the production of oestradiol by a granulosa cell tumour, is the leading cause of the development of endometrial hyperplasia in 25% to 50% of the cases, of which 5% to 13% are associated with highly differentiated early-stage endometrial carcinoma type I [6]. A retrospective analysis by Zanagnolo, V. et al., including 63 cases of stromal tumours of the genital cord treated over a 22-year period, showed that the precursor lesion of endometrial hyperplasia was diagnosed more often (26.5% of the cases) compared to endometrial carcinoma, which was diagnosed in only 8% of the cases [10].

EP are benign neoplasms of the endometrium that originate from the stroma and glands. The aetiology and pathogenesis of these polyps are unknown [11]. EP undergo malignant transformation under the influence of risk factors such as advanced age, hyperestrogenemia, postmenopausal bleeding, obesity, and the use of tamoxifen in a wide range of up to 12.9% [4,5]. In the clinical case we present, the oestrogen-secreting tumour, postmenopausal age, the presence of abnormal uterine bleeding, and obesity are the leading risk factors for the malignancy of an endometrial polyp and the development of atypical hyperplasia of the endometrium. These are the most common precancerous lesions before endometrial carcinoma type I [5]. According to Dr. Bokhman’s classification, endometrial carcinoma is separated into type I and type II carcinoma. In a hyperestrogenic environment, from precursor lesions as a multistage progression, the more common type I endometrioid adenocarcinoma, associated with a good prognosis for the patient, arises. Type II non-endometrioid carcinoma arises de novo without a precursor lesion and is oestrogen-independent as a heterogeneous group of histologic types, e.g., serous carcinoma, and correlates with a poorer prognosis [3].

Lin, Y.K. et al. described a case of a postmenopausal woman with an ovarian granulosa cell tumour and genital bleeding, who was diagnosed with stage IB invasive endometrioid adenocarcinoma, illustrating the progression of the malignant process under the influence of hyperestrogenemia [12]. Ukah, C. et al. reported a similar case, which confirms that postmenopausal women with a granulosa cell tumour can be diagnosed with early stages of endometrioid adenocarcinoma with a good prognosis [13]. Antunes, A. et al. determined that the mean age of women with malignant polyps is 64.4 ± 10.4 years [14]. Goncharenko, V.M. et al. showed that in patients with glandular hyperplasia, endometrial oestrogen receptors (ERs) are expressed to a higher degree in the epithelium, endometrial polyps, and stroma. In atypical hyperplasia, progesterone receptors (PRs) are significantly increased in the stroma, and Ki-67 expression is increased in the epithelium without changes in the stroma [15].

De Carvalho, S. et al. studied thirty postmenopausal women with benign EPs by analysing the immunoexpression of the ER and PR in the glands and stromal cells of the polyps. The results obtained indicated a high proportion of steroid hormone receptors in the glands of the polyps, which is evidence of their leading role in the etiopathogenesis of endometrial neoplasms [16]. In the glandular epithelium of endometrial polyps, the immunohistochemical detection of ER and PR is higher compared to the surrounding endometrium. Based on these results, Sant’Ana de Almeida, E. C and colleagues suggested that steroid hormone receptors play a key role in the pathogenesis of endometrial polyps. Polyps in postmenopausal women and ER have a specific influence [17].

Other authors reported that the ER expression in the stroma of benign ER was higher than that in malignant endometrial polyps, and no difference was found in PR expression. It follows that the malignant transformation of the endometrial polyp is also associated with low stromal ER expression, but the PR expression showed no association with the risk of malignancy [14]. It is of utmost importance to know the pattern of steroid receptors in human endometrial tissue, because this may open a new field in the hormonal therapy of endometrial cancer [18].

Other studies, such as that of Daniilidou, K. et al., emphasise the role of p-53 as a marker for early type II endometrial carcinoma, which could also develop in an endometrial polyp [19].

Abrão, F. et al. conducted a retrospective study on PTEN and p-53 protein immunoexpression and the risk of malignancy in endometrial polyps. In the case with p53+ and PTEN, malignant transformation was more common [20]. In comparison, p-53 was negative in the presented case. P-53 expression in atypical endometrial hyperplasia is low or absent according to Niu, S. et al. [21]. This may be explained by the fact that p-53 is a tumour suppressor gene. Therefore, its absence of expression signifies its repression, a necessary condition for malignancy. In 2024, Nakajima, J. and colleagues published the result of an analysis of p-53 expression and HER 2 expression in endometrial carcinoma and highlighted the importance of the aberrant coexpression of the two biomarkers as an independent poor prognostic factor for early-stage endometrial carcinoma [22]. Some authors emphasise the importance of examining hormone receptors and these and other biomarkers for the accurate diagnosis of possible different endometrial carcinomas, determining the therapeutic approach and also the prognostic aspects. The morphological type and immunoprofile of an endometrial neoplasm give us certain prognostic information [19].

Ki-67 is a nuclear antigen that shows the number of cells that can enter the mitotic phase, which defines it as a marker for proliferation. In benign EP with a large number of cells in the glandular epithelium, Ki-67 is positive [23].

Ki-67 positivity in the present case was low. This was also cited by Antunes, A. et al., who evaluated the expression pattern of Ki-67, Bcl-2, and COX-2 in malignant and benign postmenopausal EP and found that there was no difference in the expression of Ki-67 in the two types of EP [24]. The overexpression of c-erbB2 in EP may lower their threshold to oestrogens, making them sensitive to the low levels encountered during menopause, while the surrounding endometrium remains atrophic and unresponsive. Maia, H. and colleagues reported a directly proportional relationship between c-erbB2 overexpression in the polyp and the high rate of Ki-67 expression, which determined the proliferation profile of EP during menopause [23].

Another study conducted by Inceboz, U. S. et al. among women with postmenopausal abnormal bleeding diagnosed with endometrial polyps emphasises the role of oestrogen in the pathogenesis of EP, with an emphasis on the stimulation of proliferation through the significant expression of ER and PR and Ki-67 [25]. Regarding the hormonal influence, we also assume that it has a stimulating effect on the development of polyps and their malignancy. The studies by Giordano, G. et al. and Giordano, M. et al. showed that postmenopausal status and obesity are additional independent risk factors for the occurrence of carcinoma in EP in women without breast cancer. In obese women, oestrogens are produced from androgens under the influence of aromatase. The expression of oestrogen and progesterone steroid receptors in polyps is increased in obese women [26,27].

### Limitations

The urgent hospitalisation and personal considerations of the patient limited the preoperative imaging diagnosis by means of CT or MRI. The referral of the patient to another centre after the operative intervention limited the direct follow-up of the development of the clinical case.

## 4. Conclusions

The presented clinical case illustrates the diagnostic–therapeutic approach in a woman with postmenopausal uterine bleeding due to intrauterine pathology arising from an oestrogen-producing ovarian tumour. Precancerous lesions of the endometrium in combination with granulosa cell tumours of the ovaries are extremely rare. Making the correct diagnosis relies not only on clinical and imaging diagnostic methods but also on a detailed histopathological examination. Knowledge of the risk factors helps us in clinical diagnoses. The analyses of the expression of steroid hormone receptors and biomarkers reveal details and complement good diagnostic methods, allowing us to distinguish and draw conclusions about the different types of endometrial transformation. This, in turn, is strictly correlated with the prognosis for the patient in whom surgical treatment is the first choice. Postmenopausal status and obesity are additional independent risk factors for endometrial polyp malignant degeneration and the development of type I endometrial carcinoma.

## Data Availability

The data presented in this study are available on request from the corresponding author. The data are not publicly available due to ethical restrictions.
